# Quantitative Analysis of Flavonoids in Fruiting Bodies of *Sanghuangporus* Using Ultra-High-Performance Liquid Chromatography Coupled with Triple Quadrupole Mass Spectrometry

**DOI:** 10.3390/molecules28135166

**Published:** 2023-07-02

**Authors:** Zhongjing Zhou, Zhiping Deng, Shuang Liang, Xiaowei Zou, Yi Teng, Weike Wang, Lizhong Fu

**Affiliations:** 1 State Key Laboratory for Managing Biotic and Chemical Threats to the Quality and Safety of Agro-Products, Zhejiang Academy of Agricultural Sciences, Hangzhou 310021, China; zhouzj@zaas.ac.cn (Z.Z.); liangs@zaas.ac.cn (S.L.); tengy@zaas.ac.cn (Y.T.); 2 Institute of Virology and Biotechnology, Zhejiang Academy of Agricultural Sciences, Hangzhou 310021, China; zhipingdeng@zaas.ac.cn; 3 College of Pharmaceutical Science, Zhejiang Chinese Medical University, Hangzhou 311402, China; zouxiaowei@zcmu.edu.cn; 4 Hangzhou Academy of Agricultural Sciences, Hangzhou 310024, China

**Keywords:** flavonoids, UHPLC-MS/MS, *Sanghuangporus*, quantitative

## Abstract

A rapid, precise, and dependable method for quantifying flavonoids in the fruiting bodies of *Sanghuangporus* was established using ultra-high-performance liquid chromatography coupled with triple quadrupole mass spectrometry (UHPLC-QQQ-MS/MS). Separation was achieved using a ZORBAX Eclipse Plus C18 column (1.8 μm, 3.0 mm × 100 mm) with a 15 min gradient of a mobile phase consisting of 0.01% aqueous formic acid and 2 mm/L ammonium formate (mobile phase A), and 0.01% formic acid and 2 mm/L ammonium formate in methanol (mobile phase B). A mass spectrometry analysis was performed using the multiple reaction monitoring (MRM) mode with an electrospray ion source. This method enabled the simultaneous detection of 10 flavonoids (sakuranetin, quercitrin, myricitrin, kaempferol, luteolin, rutin, hyperoside, kaempferol-3-*O*-rutinoside, catechin, and catechin gallate) in the fruiting bodies of *Sanghuangporus*. Additionally, we applied this method to analyze the flavonoid content in fruiting bodies of various *Sanghuangporus* species. The results revealed substantial variations in flavonoid content, up to a 100-fold difference, among different species, with myricitrin, hyperoside, and rutin identified as the most abundant flavonoids. This protocol serves as a valuable tool for quantifying flavonoid compounds in different *Sanghuangporus* species or under diverse cultivation conditions, particularly for identifying species with high levels of specific flavonoid compounds.

## 1. Introduction

The Sanghuang mushroom, also known as *Sanghuangporus*, is a rare and perennial medicinal mushroom belonging to the Basidiomycota fungal phylum, Agaricomycetes class, Hymenochaetales order, and Hymenochaetaceae family [1]. It is referred to as “Sanghuang” in China, “Sanghwang” in Korea, and “Meshimakobu” in Japan [2,3]. In ancient Chinese medical classics such as *Shennong’s Classic of Materia Medica* (Shennong Bencao Jing), *Theory of Medicinal Properties* (Yaoxing Lun), *Newly Revised Materia Medica* (Xinxiu Bencao), and *Compendium of Materia Medica* (Bencao Gangmu), the Sanghuang mushroom is also known as “Sang’er” or “Sangchen”. *Sanghuangporus* has been traditionally used in Chinese medicine to treat various health conditions such as night sweats, dysentery, blood strangury, rectal prolapse and anorectal bleeding, astringent pain of the umbilicus and abdomen, amenorrhea, gynecological diseases, and spleen deficiency diarrhea [4]. Modern pharmacological research has confirmed its therapeutic effects, including antitumor [5,6], immunomodulatory [7], anti-inflammatory [8], antioxidant [1,9,10], anti-influenza [11], antidiabetic, antiangiogenic, radioprotective, and hypoglycemic [5,12,13] effects. In addition, *Sanghuangporus* is known to inhibit the growth of various malignant tumors, including ascite tumors, breast cancer, and gastric cancer, making it an exceptionally effective antitumor agent among giant fungi worldwide [14]. Considering its high medicinal and commercial value, *Sanghuangporus* has attracted the attention of many researchers. Due to the increased market demand and slow natural growth of *Sanghuangporus* mushrooms, artificial cultivation of their fruiting bodies has been a focus since the 1990s [15].

Flavonoids are secondary metabolites found in plants and medicinal fungi. They exhibit various pharmacological activities, such as antitumor, antioxidant, anti-inflammatory, and antidiabetic effects [16]. Liu et al. used an LC-MS-IT-TOF system and revealed the presence of flavonoids in fruiting bodies, including isorhamnetin, quercitrin, rutin, and quercetin [17]. Mushrooms do produce flavonoids, as Wang et al. recently used metabolomics and transcriptome analyses and confirmed the flavonoid biosynthetic pathway in medicinal mushrooms [18]. *Sanghuangporus* has been shown to contain flavonoids and is characterized by a high content [19], with various biological activities including antioxidant [20], antitumor [21], and α-amylase inhibitory properties [20]. Flavonoids identified in *Sanghuangporus* include glycosylated or acylated genistein, pinobanksin, and kaempferol, which have been studied as anticancer agents [18]. Studies have shown that the antioxidant capacity of *Sanghuangporus* is mainly correlated with the content of flavonoids and ascorbic acid [10]. Kaempferol is a natural antioxidant that protects the heart from disease damage through anti-apoptotic, anti-inflammatory, calcium-regulatory, and anti-fibrotic mechanisms [22]. Luteolin has anti-inflammatory, antioxidant, and immune-regulatory activity and has long been used in Asian medicine to treat various human diseases [23]. Rutin is a polyphenolic flavonoid with anti-inflammatory and antioxidant properties and is of significant medical importance [24]. The quantitative analysis of active ingredients in *Sanghuangporus* is crucial for evaluating its quality, and identifying antioxidants from it can enhance the accuracy of product quality assessment [25]. Therefore, searching for potential biomarkers in flavonoids may become a crucial criterion for evaluating the efficacy of *Sanghuangporus* therapy. To identify these biomarkers, it is essential to conduct qualitative and quantitative analyses of the specific contents of flavonoids. Thus, it is imperative to establish a detection method for flavonoids.

Ultra-high performance liquid chromatography (UHPLC) coupled with triple quadrupole mass spectrometry (QQQ-MS) provides accurate and sensitive results in the multiple reaction monitoring (MRM) mode, making it suitable for a quantitative analysis [26]. UHPLC-MS/MS methods have been applied to determine bioactive components in traditional Chinese medicine [27]. Song et al. employed a UPLC-MS/MS-based metabolomic analysis to establish the metabolic profile of *Sanghuangporus* basidiocarps, which serves as a valuable reference for the comprehensive evaluation and utilization of *Sanghuangporus* [28]. However, there is still a significant need for research to quantify flavonoid monomers in *Sanghuangporus* using UHPLC-QQQ-MS/MS.

In this study, a rapid, reliable, and sensitive UHPLC-MS/MS method was established using the MRM mode to simultaneously quantify 10 flavonoid compounds (sakuranetin, quercitrin, myricitrin, kaempferol, luteolin, rutin, hyperoside, kaempferol-3-*O*-rutinoside, catechin, and catechin gallate) in *Sanghuangporus*. With a small sample quantity (less than 100 mg), this method provides robust technical support for a comprehensive quality control assessment of *Sanghuangporus*. This study expands our knowledge of the quantitative and qualitative analysis of multiple flavonoids in *Sanghuangporus* and offers critical guidance for exploring other potential resources within *Sanghuangporus*. Furthermore, this method contributes to further developing and utilizing bioactive agents derived from *Sanghuangporus* species.

## 2. Results

### 2.1. Optimization of Sample Preparation Methods

**Figure 1:**
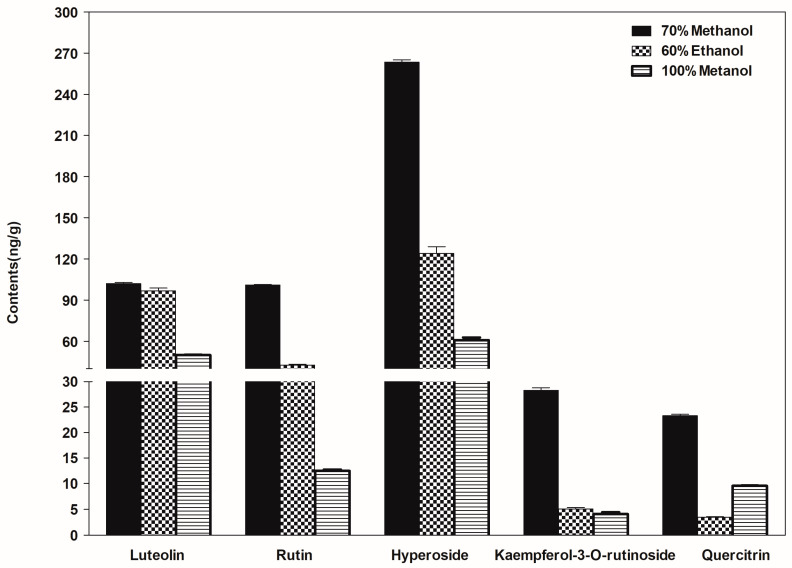
Effects of different extraction solvents on compound (luteolin, rutin, hyperoside, kaempferol-3-*O*-rutinoside, and quercitrin) content (ng/g).

The observed content of plant metabolite results depends highly on the extraction conditions [26]. To achieve optimal pretreatment of *Sanghuangporus*, methanol and ethanol were selected as extraction solutions at varying concentrations (70% methanol, 60% ethanol, and 100% methanol). The fruiting body of a wild *Sanghuangporus Sanghuang*, namely H17, was used for optimization. Guo et al. [6] found that the 60% ethanol extract yielded the highest flavonoid content. In our experiment, the extraction of flavonoids from the H17 sample (Figure 1) was performed following descriptions provided by Song [28], Fu [29], and Guo [6] with some modifications. Among the three extraction solvents tested, our study found that 70% methanol was the most efficient solvent for sample extraction of the five flavonoid compounds: luteolin, rutin, hyperoside, kaempferol-3-*O*-rutinoside, and quercitrin (Figure 1). Therefore, we chose 70% methanol as the extraction solvent.

### 2.2. Optimization of MS/MS Conditions

To optimize the MS/MS parameters, a direct infusion of 100 μg/L of each standard solution was performed using an injection pump at a flow rate of 7 μL/min. The mass spectrometry response of compound ion pairs was analyzed separately in both electrospray ionization (ESI) positive and negative ion modes, considering the properties of the 10 flavonoids. The precursor ions were first identified, and then the most intense product ions were selected based on the fragmentation patterns of the precursor ions. All analytes showed a maximum sensitivity in the negative; hence, the negative ESI mode was chosen for the analysis. The retention time, decluttering potential (DP), and collision energy (CE) for each analyte were determined, and the values are shown in Table 1.

### 2.3. Optimization of Chromatography Conditions

**Figure 2:**
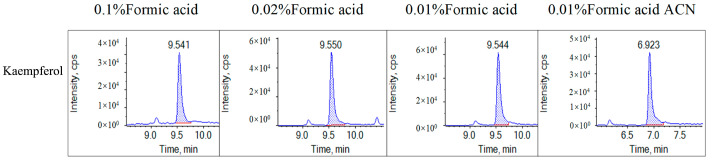
The influence of different concentrations of formic acid on the responses and peak shapes of the kaempferol. The aqueous phase contains 0.1% formic acid, 0.02% formic acid, and 0.01% formic acid, respectively; the organic phase is methanol. The label of 0.01% formic acid ACN means that the aqueous phase contains a 0.01% formic acid aqueous solution, and the organic phase is acetonitrile.

Optimizing the mobile phase was conducted to achieve the best separation and a good chromatographic shape. Adding appropriate amounts of formic acid and ammonium formate to the mobile phase can impact the responses and peak shapes of the selected analytes, as noted by Zhao et al. [26]. To determine the optimal conditions for our experiment, we compared the impact of 0.1%, 0.02%, and 0.01% formic acid in aqueous solutions on kaempferol’s peak height and shape. Our results revealed that the 0.01% formic acid aqueous solution produced kaempferol’s best peak height and shape (Figure 2).

**Figure 3:**
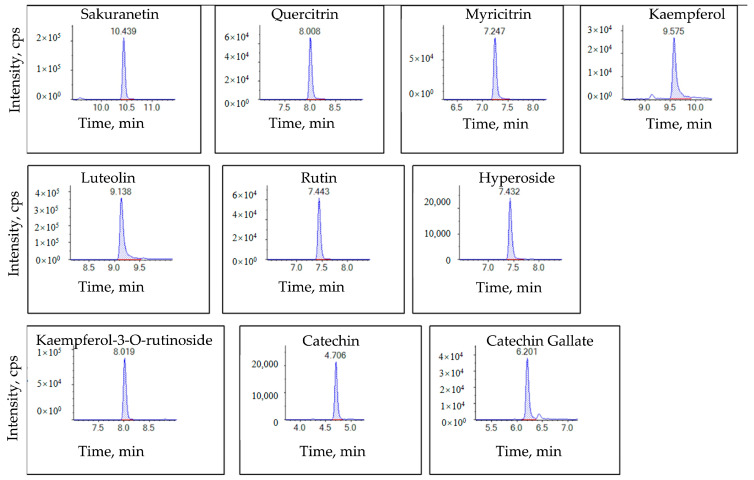
The extracted ion chromatogram (XIC) of the 10 flavonoid standards (sakuranetin, quercitrin, myricitrin, kaempferol, luteolin, rutin, hyperoside, kaempferol-3-*O*-rutinoside, catechin, and catechin gallate); the working concentration of each compound was 100 μg/L.

When acetonitrile was used in the organic phase, we observed a severe chromatographic peak trailing in the kaempferol (Figure 2). However, when we used an organic phase containing formic acid and ammonium formate in methanol, we observed an improved separation capacity, peak shape, and signal intensity (Figure 3). Therefore, we chose the 0.01% formic acid and 2 mmol/L ammonium formate aqueous solution as mobile phase A and the 0.01% formic acid and 2 mmol/L ammonium formate methanol solution as mobile phase B.

### 2.4. Validation of the Method

#### 2.4.1. Linearity, Limit of Detection (LOD), and Limit of Quantification (LOQ)

Mixed standard working solutions were used in at least five concentrations (0.05 μg/L, 0.1 μg/L, 0.5 μg/L, 1 μg/L, 5 μg/L, 10 μg/L, 50 μg/L, and 100 μg/L) to prepare the calibration curve for each compound. The mass concentration was plotted on the horizontal axis, and the peak area on the vertical axis, to generate the standard curve. The limits of detection (LOD) and quantification (LOQ) were then calculated at signal-to-noise (S/N) ratios of 3 and 10, respectively. Table 2 presents detailed information on calibration curves, linear ranges, LOD, and LOQ. Results indicated that each analyte demonstrated a good linearity, with coefficients of determination (R^2^) greater than 0.999. These findings suggest that the sensitivity of the current method is sufficient for determining these compounds in the fruiting bodies of *Sanghuangporus*.

#### 2.4.2. Precision

To validate the precision of the established method, we conducted tests on both intra-day and inter-day variances. For the intra-day test, samples were injected six times within 1 day, while for the inter-day test, samples were examined over 6 consecutive days. The concentration of each solution was determined with a calibration curve formed on the same day. The intra- and inter-day precisions, calculated as a relative standard deviation (RSD), are presented in Table 2. The precision values for intra-day and inter-day tests ranged from 0.9–15.4% and 1.0–18.3%, respectively, indicating that our method has a good repeatability and reproducibility.

### 2.5. Target Flavonoid Compound Analysis in Fruiting Bodies of Sanghuangporus

**Figure 4:**
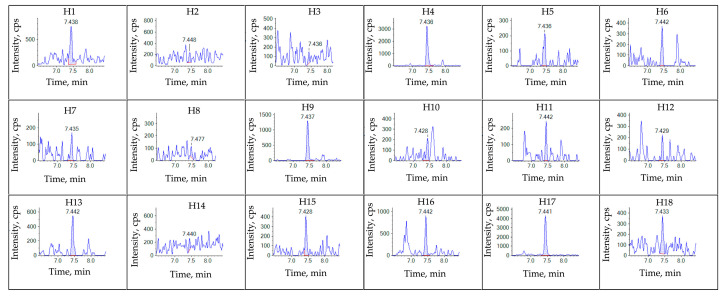
The extracted ion chromatogram (XIC) of the rutin in the 18 fruiting bodies of Sanghuangporus. Samples: H1–H18.

The developed and validated method was applied to detect and quantify the 10 selected flavonoids in 18 samples of *Sanghuangporus* (Table 3). The quantitative results of the quercitrin, myricitrin, kaempferol, luteolin, rutin, hyperoside, and kaempferol-3-*O*-rutinoside in each sample are presented in Table 4. There were extremely low concentrations detected for the other three compounds, sakuranetin, catechin, and catechin gallate. Based on the sample results, it is evident that *Sanghuangporus* contains relatively low amounts of myricitrin, kaempferol, luteolin, hyperoside, and kaempferol-3-*O*-rutinoside. In contrast, the levels of rutin and quercitrin are relatively high. Rutin is often used as a standard substance for determining total flavonoid content. The extracted mass spectrum (XIC) area of rutin in 18 samples can be seen in Figure 4. Sample H11 especially showed a range of 8.5 ng/g, but the chromatographic separation effect was still good, indicating the high sensitivity of this method. This method is believed to provide strong technical support for further research on active flavonoid markers in *Sanghuangporus* research.

## 3. Discussion

*Sanghuangporus* is a medicinal mushroom commonly used in traditional Chinese medicine. Flavonoids are key bioactive compounds found in *Sanghuangporus*, and the quantification of flavonoid active substances is important for the identification and quality control of this mushroom [18]. Shen et al. reported that the main antioxidant components in *S. baumii* are flavonoids and polyphenols. They used OLE-HPLC-ABTS technology to establish an online screening method to rapidly discover the antioxidants in *S. baumii* [25]. However, more reliable methods need to be used to quantify flavonoids in *Sanghuangporus*.

Our study developed a rapid and sensitive UHPLC-QQQ-MS method for simultaneously analyzing 10 flavonoid monomers in *Sanghuangporus*. This analytical method enables the quick detection and quantification of flavonoids in the fruiting bodies of *Sanghuangporus* with a high accuracy, precision, and selectivity. Our advanced UHPLC-QQQ-MS analytical method provides several advantages over previously reported methods for quantifying flavonoid monomers in *Sanghuangporus*. Firstly, it is more sensitive and selective since it can detect 10 flavonoid monomers simultaneously. Secondly, by using a C18 column and optimizing the mobile phase, it has an excellent separation efficiency, which is crucial for accurately quantifying flavonoid monomers. Finally, our method is rapid, simple, and easy to use, which makes it suitable for a routine analysis of large sample numbers. Our method is not only a valuable tool for identifying and controlling the quality of *Sanghuangporus*. It can also be used to screen *Sanghuangporus* varieties with higher contents of specific flavonoid monomers. Therefore, our method contributes to the utilization and quality control of *Sanghuangporus* as a natural health product. This method will be an important tool for comparing flavonoid compounds in different *Sanghuangporus* species, particularly for screening those with higher content of specific flavonoid monomers.

Flavonoids are widely present in plants, such as vegetables and fruits. These compounds are classified into seven subgroups: flavanol, flavone, isoflavone, flavanone, anthocyanin, chalcone, and flavonol [22]. Our results indicate that the contents of kaempferol, luteolin, and rutin in a wild-grown *Sanghuangporus* sample were at least five-fold higher than most other artificially cultivated *Sanghuangporus* samples (Table 4). This suggests that our detection method is practical and effective and can provide technical support for the future artificial cultivation of *Sanghuangporus*.

This study established a fast detection method of flavonoids in *Sanghuangporus* using UHPLC-MS/MS, which has the advantages of an extremely low sample consumption, higher analytical efficiency, and sensitive detection. The rapid quantification of flavonoids, as the main antioxidant components in *Sanghuangporus*, can be used as markers for the quality control of *Sanghuangporus* and its products. In the future, analytical methods for determining these antioxidants should be developed to analyze different *Sanghuangporus* samples. Moreover, due to the low sample consumption (mg level), this method is more suitable for analyzing active ingredients in valuable medicinal materials. In conclusion, as a green analytical chemistry method with little pollution, it provides a possible developmental direction for the analysis and quality control of the *Sanghuangporus* species and the discovery of bioactive compounds.

## 4. Materials and Methods

### 4.1. Materials

The samples used in this research were fruiting bodies of *Sanghuangporus vaninii*, *Sanghuangporus baumii*, and *Sanghuangporus. sanghuang*. The samples H1–H8 and H14 belong to *S. baumii*; H9–H13, H16, and H18 belong to *S. vaninii*; H15, and H17 is from *S. sanghuang*, detailed information can be seen in Table 3. The *S. vaninii*, *S. baumii*, and *S. sanghuang* strains were identified with precision, utilizing their morphological features and internal transcribed spacer sequencing. The NCBI Accession Nos. MN153566, MN153567, and MN153568 were used for their identification, respectively. These strains were subsequently preserved at the Hangzhou Academy of Agricultural Sciences.

### 4.2. Chemicals and Reagents

LC-MS-grade methanol was purchased from Merck (Darmstadt, Germany). Formic acid was purchased from Fisher Scientific (LC-MS grade, Thermo Scientific, Carlsbad, CA, USA), and ammonium formate was obtained from Macklin (HPLC grade, purity: ≥99%). Sakuranetin was purchased from Sigma-Aldrich (Sigma-Aldrich, Inc., St. Louis, MO, USA, CAS number: 2957-21-3); quercitrin (CAS number: 522-12-3), myricitrin (CAS number: 17912-87-7), kaempferol (CAS number: 520-18-3), luteolin (CAS number: 491-70-3), rutin (CAS number: 153-18-4), hyperoside (CAS number: 482-36-0), kaempferol-3-*O*-rutinoside (CAS number: 17650-84-9), catechin (CAS number: 225937-10-0), and catechin gallate (CAS number: 130405-40-2) were purchased from Shanghai Yuanye Bio-Technology Co., Ltd. (Shanghai, China). Ultrapure water was purified using a Milli-Q system (18 MΩ*cm, Millipore, Burlington, MA, USA).

### 4.3. Instruments

The UHPLC-MS/MS system consisted of Triple Quad™ LC-MS/MS 5500+ (AB SCIEX, USA) and Ultra Performance Liquid Chromatography UHPLC, ExionLC™ AD (Shimadzu, Kyoto, Japan). A Xinzhi SB25-12DTD ultrasonic bath (Ningbo, China) was used for the supersonic-assisted extraction. A Vortex-Genie 2 (Scientific Industries, Bohemia, NY, USA) was employed to mix the sample solution. A heating and drying oven (Jinghong DHG-9240A, Shanghai, China) was used for drying and heating treatment of samples. An electronic analytical balance (Mettler-Toledo ME204, Zürich, Switzerland) was used to weigh samples precisely. A medical refrigerator (Aucma YCD-265, Qingdao, China) was used for storing experimental samples. A 0.22 μm syringe filter (Biosharp life sciences, Hefei, China) was used for sample filtration. Centrifugation of the sample solution was accomplished with a 5418 high-speed centrifuge (Eppendorf Corp., Hamburg, Germany).

### 4.4. Preparation of Standard Solutions

Each compound was accurately weighed and dissolved in methanol to prepare a standard stock solution at 1 mg/mL concentrations. Subsequently, the standard working solutions of the analytes were prepared by diluting the mixed stock solution with 70% methanol–water (*v*/*v*) to the required concentration. Various working solutions with different mass concentrations were produced and stored for future usage under dark conditions in a −20 °C refrigerator.

### 4.5. Sample Preparation

The *Sanghuangporus* sample was ground into dry powder and passed through an 80-mesh sieve. Six aliquots of 0.1 g *Sanghuangporus* powder were accurately weighed and placed in 2 mL centrifuge tubes, respectively. Then, 1.5 mL of 70% methanol–water (*v*/*v*) was added to each tube and vortexed for 5 min, followed by extraction in an ultrasonic bath for 30 min. The mixture was centrifuged at 12,000 r/min for 20 min at 4 °C, and the supernatant was filtered through a 0.22 μm syringe filter before injection into the UHPLC-MS/MS system for the analysis.

### 4.6. UHPLC Conditions

The liquid phase separation was performed on the ZORBAX Eclipse Plus C18 column (1.8 μm, 3.0 mm × 100 mm, Agilent Technologies Inc., Santa Clara, CA, USA) at a column temperature of 40 °C. The mobile phase included a 0.01% formic acid and 2 mmol/L ammonium formate water solution (Buffer A) and 0.01% formic acid and 2 mmol/L of ammonium formate in methanol (Buffer B). The gradient elution program was optimized as follows: 0~1.0 min, 90% A, 10% B; 1.0~11.0 min, 90% A~5% A, 10% B~95% B; 11.0~13.0 min, 5% A, 95% B; 13.0~13.1 min, 5% A~90% A, 95% B~10% B; and 13.1~15 min, 90% A, 10% B. The flow rate was 0.4 mL/min, and the injection volume was 1 μL. 

### 4.7. MS/MS Conditions

The mass spectrometry data were collected using the dynamic MS/MS acquisition software Analyst 1.7.1, and SCIEX OS software (https://sciex.com.cn/products/software/sciex-os-software (accessed on 15 June 2023)) was used for the data analysis. The ion source was operated in the negative electrospray ionization mode (ESI−) with multiple reaction monitoring (MRM) scans. The spray voltage was −4500 V under the negative ion mode, and the ion source temperature was 500 °C. Ion Source Gas1 (Gas1) pressure was 50 psi, and the auxiliary gas (Ion Source Gas2, Gas2) pressure was 55 psi. Curtain Gas (CUR) pressure was 35 psi, and Collision Gas (CAD) pressure was 8 psi. The scanning and detection parameters for each ion pair based on the optimized clustering potential (DP) and collision energy (CE) are detailed in Table 1.

### 4.8. Statistical Analysis

Quantitative data are presented as mean values with a standard deviation of the means derived from six independent experiments. The data were analyzed using Microsoft Excel. GraphPad Prism 5.0 software (GraphPad Software, San Diego, CA, USA) was used to analyze the data and construct the graphical outputs shown in Figure 1.

## 5. Conclusions

This study aimed to (1) establish an ultra-high-performance liquid chromatography with triple quadrupole mass spectrometry (UHPLC-QQQ-MS/MS) method for the simultaneous detection of 10 flavonoids in *Sanghuangporus*; (2) evaluate the linearity, limit of detection (LOD), limit of quantification (LOQ), accuracy, and precision of the method; and (3) use the developed method to measure the content of flavonoids in 18 samples of *Sanghuangporus*. There is a need for further basic research and development of detection methods for flavonoid-active substances in *Sanghuangporus*. This method will advance the study of the composition and distribution of flavonoids in *Sanghuangporus* and provide strong theoretical support for developing *Sanghuangporus* as a new food and health food resource and for related product research and development.

## Figures and Tables

**Table 1:** Retention time (RT) and MRM conditions of 10 compounds for UHPLC-ESI-MS/MS analysis.

Analyte	Q1	Q3	RT (min)	DP (V)	CE (V)
Sakuranetin ^1^	285.1	119.1	10.44	−130	−42
Sakuranetin ^2^	285.1	97	10.44	−130	−31
Quercitrin ^1^	447.1	271	8.00	−76	−62
Quercitrin ^2^	447.1	255	8.00	−76	−51
Myricitrin ^1^	463.1	316	7.25	−72	−35
Myricitrin ^2^	463.1	271	7.25	−76	−52
Kaempferol ^1^	285.1	143	9.58	−130	−43
Kaempferol ^2^	285.1	117.1	9.58	−130	−50
Luteolin ^1^	285.1	133.2	9.14	−115	−46
Luteolin ^2^	285.1	132.1	9.14	−115	−65
Rutin ^1^	609.2	300.1	7.44	−120	−48
Rutin ^2^	609.2	271	7.44	−120	−70
Hyperoside ^1^	463.2	301.2	7.43	−129	−33
Hyperoside ^2^	463.2	150.9	7.43	−100	−42
Kaempferol-3-*O*-rutinoside ^1^	593.2	285.1	8.02	−100	−43
Catechin ^1^	289.1	109.1	4.71	−118	−37
Catechin ^2^	289.1	123	4.71	−120	−39
Catechin Gallate ^1^	441.1	169.2	6.20	−130	−30

^1,2^ refers to the information on each compound’s parent and daughter ions.

**Table 2:** Linear regression, LOD, LOQ, Intra-day and Inter-day for flavonoids (Sakuranetin, Quercitrin, Myricitrin, Kaempferol, Luteolin, Rutin, Hyperoside, Kaempferol-3-*O*-rutinoside, Catechin, and Catechin Gallate) in *Sanghuangporus*.

Analyte	Regression Equation	R^2^	Linear Range (μg/L)	LOD ^1^ (μg/L)	LOQ ^2^ (μg/L)	Intra-Day (RSD, %) (*n* = 6)	Inter-Day (RSD, %) (*n* = 6)
Sakuranetin	y = 7047.30x − 397.62	0.99999	0.50–100	0.100	0.333	2.5–3.7	6.1–7.2
Quercitrin	y = 2524.34x − 373.40	0.99965	0.50–100	0.273	0.909	1.1–8.9	1.0–12.5
Myricitrin	y = 3442.19x − 366.26	0.99974	0.50–100	0.429	1.429	3.1–12.6	5.9–17.7
Kaempferol	y = 827.16x + 56.89	0.99998	1.00–100	0.857	2.857	2.1–15.4	1.5–5.9
Luteolin	y = 18,920.86x − 102.42	0.99905	0.05–100	0.009	0.029	0.9–3.7	1.6–4.5
Rutin	y = 1248.26x − 187.98	0.99953	0.50–100	0.333	1.111	3.8–7.2	3.7–17.2
Hyperoside	y = 1003.12x − 61.44	0.99946	1.00–100	0.462	1.538	3.0–10.4	2.5–18.3
Kaempferol-3-*O*-rutinoside	y = 3572.55x − 1111.41	0.99968	0.50–100	0.273	0.909	1.7–5.7	3.6–6.1
Catechin	y = 867.22x + 167.18	0.99903	0.10–100	0.086	0.286	2.3–9.2	3.4–14.0
Catechin Gallate	y = 1485.81x − 62.16	0.99917	0.50–100	0.667	2.222	2.3–4.2	1.9–7.8

^1^ LOD, Limit of detection; ^2^ LOQ, Limit of quantification.

**Table 3:** Detailed information and main characteristics on samples of the fruiting bodies of *Sanghuangporus* species used in the present study.

Sample No.	Species	Strain	Cultivation Methods ^1^	Developmental Stages ^2^
H1	*Sanghuangporus baumii*	S13	Cut-log	1-year-old
H2	*S. baumii*	S13	Cut-log	2-year-old
H3	*S. baumii*	S13	Cut-log	3-year-old
H4	*S. baumii*	S13	Sawdust	30 days
H5	*S. baumii*	S13	Sawdust	45 days
H6	*S. baumii*	S13	Sawdust	60 days
H7	*S. baumii*	S13	Sawdust	75 days
H8	*S. baumii*	S13	Sawdust	90 days
H9	*Sanghuangporus vaninii*	S943	Sawdust	30 days
H10	*S. vaninii*	S943	Sawdust	45 days
H11	*S. vaninii*	S943	Sawdust	60 days
H12	*S. vaninii*	S943	Sawdust	75 days
H13	*S. vaninii*	S943	Sawdust	90 days
H14	*S. baumii*	S13	Cut-log	3-year-old
H15	*S. sanghuang*	S23	Cut-log	3-year-old
H16	*S. vaninii*	S19	Cut-log	3-year-old
H17	*S. sanghuang*	Wild	Wild	Wild
H18	*S. vaninii*	S93	Sawdust	90 days

^1^ Cultivation methods: Cut-log refers to a traditional cultivation method of *Sanghuangporus*, in which the mushroom spawn is inoculated into the felled logs to ferment and grow into fruiting bodies, which takes 3–4 years; sawdust cultivation refers to the cultivation of *Sanghuangporus* using sawdust as the substrate. ^2^ Collectively, 30 days, 45 days, 60 days, 75 days, and 90 days refer to the time from the formation of primordia to the harvest of fruiting bodies, which are 30 days, 45 days, 60 days, 75 days, and 90 days, respectively.

**Table 4:** Contents (ng/g) of target analytes (Quercitrin, Myricitrin, Kaempferol, Luteolin, Rutin, Hyperoside, and Kaempferol-3-*O*-rutinoside) in the fruiting bodies of *Sanghuangporus*. Values represent means ± SD (standard deviation, *n* = 6) of the samples.

Sample	Quercitrin	Myricitrin	Kaempferol	Luteolin	Rutin	Hyperoside	Kaempferol-3-*O*-Rutinoside
H1	3.99 ± 0.03	311.04 ± 1.74	3.83 ± 0.19	2.89 ± 0.08	25.398 ± 0.21	386.72 ± 4.79	14.16 ± 0.34
H2	6.07 ± 0.12	293.99 ± 5.07	8.97 ± 0.25	0.9 ± 0.04	20.23 ± 0.29	342.72 ± 1.31	8.33 ± 0.24
H3	16.28 ± 0.13	264.85 ± 2.44	1.45 ± 0.03	2.16 ± 0.02	11.79 ± 0.09	290.73 ± 3.5	13.71 ± 0.17
H4	3.42 ± 0.05	52.65 ± 0.41	6.49 ± 0.05	0.53 ± 0.01	106.25 ± 0.49	6.58 ± 0.15	15.04 ± 0.2
H5	3.23 ± 0.04	60.87 ± 0.45	2.08 ± 0.02	0.59 ± 0.01	6.57 ± 0.1	36.19 ± 0.38	7.78 ± 0.07
H6	4.12 ± 0.05	66.14 ± 0.3	14.45 ± 0.07	0.3 ± 0.01	8.72 ± 0.16	17.94 ± 0.58	12.02 ± 0.21
H7	2.76 ± 0.03	15.2 ± 0.12	4.66 ± 0.19	0.54 ± 0.01	3.56 ± 0.05	6.26 ± 0.28	24.15 ± 0.38
H8	3.13 ± 0.05	10.39 ± 0.1	4.63 ± 0.13	0.55 ± 0.003	3.35 ± 0.04	4.28 ± 0.09	13.55 ± 0.15
H9	2.43 ± 0.01	30.15 ± 0.43	4.18 ± 0.23	0.44 ± 0.01	35.77 ± 0.37	8.1 ± 0.34	9.76 ± 0.1
H10	2.67 ± 0.02	80.91 ± 0.44	12.5 ± 0.35	0.88 ± 0.005	6.46 ± 0.08	17.02 ± 0.59	6.28 ± 0.08
H11	3.14 ± 0.02	40.683 ± 0.36	7.51 ± 0.09	0.62 ± 0.01	8.76 ± 0.1	8.86 ± 0.16	8.54 ± 0.09
H12	3.12 ± 0.02	5.73 ± 0.06	5.81 ± 0.26	0.35 ± 0.01	4.96 ± 0.06	2.12 ± 0.03	14.88 ± 0.28
H13	3.26 ± 0.06	2.86 ± 0.02	2.31 ± 0.02	0.21 ± 0.003	15.08 ± 0.12	5.53 ± 0.22	15.41 ± 0.23
H14	3.93 ± 0.06	313.15 ± 4.76	10.54 ± 0.09	0.27 ± 0.01	9.12 ± 0.11	355.23 ± 2.37	18.89 ± 0.34
H15	4.13 ± 0.08	85.97 ± 0.83	9.64 ± 0.04	0.3 ± 0.01	3.55 ± 0.04	21.19 ± 0.43	6.05 ± 0.05
H16	4.17 ± 0.05	278.71 ± 2.53	3.84 ± 0.03	1.25 ± 0.02	18.02 ± 0.21	191.66 ± 1.95	7.74 ± 0.11
H17	3.5 ± 0.08	141.08 ± 0.9	64.92 ± 1.06	180.97 ± 0.99	145.6 ± 0.99	271.68 ± 3.09	26.36 ± 0.11
H18	3.76 ± 0.07	2.9 ± 0.07	6.96 ± 0.06	4.37 ± 0.13	15.68 ± 0.39	9.66 ± 0.19	6.00 ± 0.05

## Data Availability

Fruiting bodies of *Sanghuangporus* used in this study were kindly provided by Weike Wang (Hangzhou Academy of Agricultural Sciences, Hangzhou, Zhejiang, China).

## References

[1] Cai C., Ma J., Han C., Jin Y., Zhao G., He X. (2019). Extraction and antioxidant activity of total triterpenoids in the mycelium of a medicinal fungus, *Sanghuangporus sanghuang*. Sci. Rep..

[2] Zhou L.-W., Vlasák J., Decock C., Assefa A., Stenlid J., Abate D., Wu S.-H., Dai Y.-C. (2016). Global diversity and taxonomy of the Inonotus linteus complex (Hymenochaetales, Basidiomycota): *Sanghuangporus* gen. nov., *Tropicoporus excentrodendri* and *T. guanacastensis* gen. et spp. nov., and 17 new combinations. Fungal Divers..

[3] Wu S.H., Dai Y.C. (2020). Species clarification of the medicinal fungus Sanghuang. Mycosystema.

[4] Shao Q., Yang Y., Li T.T., Feng J., Liu Y.F., Yan M.Q., Tan Q. (2014). Biological activities of Inonotus baumii mycelium extract obtained by different cultivation methods. Mycosystema.

[5] Cheng J., Song J., Wei H., Wang Y., Huang X., Liu Y., Lu N., He L., Lv G., Ding H. (2020). Structural characterization and hypoglycemic activity of an intracellular polysaccharide from *Sanghuangporus sanghuang* mycelia. Int. J. Biol. Macromol..

[6] Guo S., Duan W., Wang Y., Chen L., Yang C., Gu X., Xue Q., Li R., Zhang Z. (2022). Component Analysis and Anti-Colorectal Cancer Mechanism via AKT/mTOR Signalling Pathway of Sanghuangporus vaninii Extracts. Molecules.

[7] Liu M.M., Zeng P., Li X.T., Shi L.G. (2016). Antitumor and immunomodulation activities of polysaccharide from Phellinus baumii. Int. J. Biol. Macromol..

[8] Zuo K., Tang K., Liang Y., Xu Y., Sheng K., Kong X., Wang J., Zhu F., Zha X., Wang Y. (2021). Purification and antioxidant and anti-Inflammatory activity of extracellular polysaccharopeptide from sanghuang mushroom, Sanghuangporus lonicericola. J. Sci. Food Agric..

[9] Zheng N., Ming Y., Chu J., Yang S., Wu G., Li W., Zhang R., Cheng X. (2021). Optimization of Extraction Process and the Antioxidant Activity of Phenolics from Sanghuangporus baumii. Molecules.

[10] Wang H., Ma J.X., Wu D.M., Gao N., Si J., Cui B.K. (2023). Identifying Bioactive Ingredients and Antioxidant Activities of Wild Sanghuangporus Species of Medicinal Fungi. J. Fungi.

[11] Hwang B.S., Lee I.K., Choi H.J., Yun B.S. (2015). Anti-influenza activities of polyphenols from the medicinal mushroom Phellinus baumii. Bioorg. Med. Chem. Lett..

[12] Huang Z., Liu Y., Liu X., Chen K., Xiong W., Qiu Y., He X., Liu B., Zeng F. (2022). Sanghuangporus vaninii mixture ameliorated type 2 diabetes mellitus and altered intestinal microbiota in mice. Food Funct..

[13] Lin W.C., Deng J.S., Huang S.S., Wu S.H., Chen C.C., Lin W.R., Lin H.Y., Huang G.J. (2017). Anti-Inflammatory Activity of *Sanghuangporus sanghuang* Mycelium. Int. J. Mol. Sci..

[14] Wang W.H., Yang Y., Zhu L.N., Jia W., Zhang J.S., Liu Y.F., Yan M.Q., Zhao X.L., Zhang K., Zhang H.N. (2018). Inhibitory effects of ethanol extract of *Sanghuangporus sanghuang* fruiting bodies on SW620 colon cancer cells. Mycosystema.

[15] Yang Y., Chen X.H., Dai Y.C., Zhou L.W., Cai W.M., Guo L.D., Cui B.K., Li N., Lei P., Li C.T. (2023). Sanghuang industry in China: Current status, challenges and perspectives: The Qiandao Lake declaration for sanghuang industry development. Mycosystema.

[16] Kui-Wu W., Ting-Ting Z. (2020). Bioactive Flavonoids from Verbenaceae. Mini-Rev. Org. Chem..

[17] Liu K., Xiao X., Wang J., Chen C.Y.O., Hu H. (2017). Polyphenolic composition and antioxidant, antiproliferative, and antimicrobial activities of mushroom Inonotus sanghuang. LWT Food Sci. Technol..

[18] Wang S., Liu Z., Wang X., Liu R., Zou L. (2022). Mushrooms Do Produce Flavonoids: Metabolite Profiling and Transcriptome Analysis of Flavonoid Synthesis in the Medicinal Mushroom Sanghuangporus baumii. J. Fungi.

[19] Wu S.H., Huang G.Z., Chen Y.P., Dai Y.C., Zhou L.W. (2016). Taxonomy and Development Prospects of Sanghuang (*Sanghuangporus sanghuang*). J. Fungal Res..

[20] Li T., Mei Y., Li J., Yang W., He F., Ge J., Chen F., Yang Y., Xie A., Liu Y. (2023). Comparative Compositions and Activities of Flavonoids from Nine Sanghuang Strains Based on Solid-State Fermentation and In Vitro Assays. Fermentation.

[21] Wan X., Jin X., Xie M., Liu J., Gontcharov A.A., Wang H., Lv R., Liu D., Wang Q., Li Y. (2020). Characterization of a polysaccharide from Sanghuangporus vaninii and its antitumor regulation via activation of the p53 signaling pathway in breast cancer MCF-7 cells. Int. J. Biol. Macromol..

[22] Kamisah Y., Jalil J., Yunos N.M., Zainalabidin S. (2023). Cardioprotective Properties of Kaempferol: A Review. Plants.

[23] Rocchetti M.T., Bellanti F., Zadorozhna M., Fiocco D., Mangieri D. (2023). Multi-Faceted Role of Luteolin in Cancer Metastasis: EMT, Angiogenesis, ECM Degradation and Apoptosis. Int. J. Mol. Sci..

[24] Hamad R.S. (2023). Rutin, a Flavonoid Compound Derived from Garlic, as a Potential Immunomodulatory and Anti-Inflammatory Agent against Murine Schistosomiasis mansoni. Nutrients.

[25] Shen Q.H., Huang Q., Xie J.Y., Wang K., Qian Z.M., Li D.Q. (2021). A rapid analysis of antioxidants in Sanghuangporus baumii by online extraction-HPLC-ABTS. RSC Adv..

[26] Zhao L., Zhao X., Xu Y., Liu X., Zhang J., He Z. (2021). Simultaneous determination of 49 amino acids, B vitamins, flavonoids, and phenolic acids in commonly consumed vegetables by ultra-performance liquid chromatography-tandem mass spectrometry. Food Chem..

[27] Li L., Yu X., Xie D., Peng N., Wang W., Wang D., Li B. (2022). Influence of traditional Chinese medicines on the in vivo metabolism of lopinavir/ritonavir based on UHPLC-MS/MS analysis. J. Pharm. Anal..

[28] Song J.L., Zhou Z.F., Yan J., Lu N., Cheng J.W., Yuan W.D., Wang W.K. (2021). Study of effects of bran on the metabolism of Sanghuangporus vaninii based on metabolomics of UPLC-MS/MS. Mycosystema.

[29] Fu L.Z., Lu N., Yan J., Wang W.K., Song J.L., Yuan W.D., Zhou Z.F. (2021). Analyses and evaluation of nutrition, active component and antioxidant activities of fruiting bodies of three species of Sanghuangporus. Mycosystema.

